# Smoking among inpatients in treatment for substance use disorders: prevalence and effect on mental health and quality of life

**DOI:** 10.1186/s12888-021-03252-9

**Published:** 2021-05-11

**Authors:** Lars Lien, Ingeborg Bolstad, Jørgen G. Bramness

**Affiliations:** 1 Norwegian National Advisory Unit on Concurrent Substance Abuse and Mental Health Disorders, Innlandet Hospital Trust, P.O. Box 104, 2381 Brumunddal, Norway; 2 Faculty of Social and Health Sciences, Inland Norway University of Applied Sciences, Elverum, Norway; 3 Blue Cross East, Oslo, Norway; 4 Department of Alcohol, Norwegian Institute of Public Health, Tobacco and Drugs, Oslo, Norway; 5 Institute of Clinical Medicine, UiT The Arctic University of Norway, Tromsø, Norway

**Keywords:** Mental health problems, Alcohol use disorder, Smoking, Drop-out

## Abstract

**Background:**

Smoking is still prevalent among people with substance use disorders. The objective of this study was to investigate the prevalence of smoking among patients in treatment for substance use disorders and to analyze the effect of smoking both at baseline and follow-up on drop-out, mental health and quality of life.

**Methods:**

One hundred and twenty-eight inpatients (26% female), mainly with alcohol use disorder, staying at three different rehabilitation clinics in Eastern Norway, were interviewed at admission, and at 6 weeks and 6 months follow-up. The interview contained mental health-related problems, trauma, questions on alcohol and other substances and quality of life. Non-parametric tests were used to test group differences and unadjusted and adjusted linear regression to test the associations between smoking and the main outcome variables, while logistic regression was used to test the association between smoking and drop-out.

**Results:**

At admission, 75% were daily smokers. Compared to non-smokers at baseline, the smokers had higher drop-out rates (37% vs. 13%), more mental distress, and lower quality of life from baseline up to 6 months follow-up. Those quitting smoking while admitted improved in mental distress and quality of life at the same rate as non-smokers. Alcohol-related factors did not differ between smokers and non-smokers.

**Conclusions:**

Smoking was associated with mental distress, quality of life and treatment drop-out among patients in primary alcohol use disorder treatment. The results indicate that smoking cessation should be recommended as an integral part of alcohol use treatment both before and during inpatient treatment to reduce drop-out.

## Background

Tobacco use is one of the leading preventable causes of death in the world, killing up to half of users with a particularly high toll on patients with mental health and addiction problems [1]. The number of smokers in the general population is rapidly decreasing. In 1973, 45% of the Norwegian population were daily smokers, compared to 26% in 2004 and 9% in 2020 [2]. These low figures contrast with the prevalence of smoking among people with addiction challenges, where studies from Australia show that 61% of those with alcohol use disorder (AUD) smoked compared with 22% of the general population in 2007 and that up to 77% of people in substance use dependence (SUD) treatment were smokers [3]. In another study, over a third of all smokers reported that they increased their smoking during residential treatment [4].

There are several explanations for the high sustained smoking rate among AUD patients. Studies have identified a shared genetic predisposition to nicotine and alcohol [5]. This results in both nicotine and alcohol triggering the release of dopamine in the reward pathway, possibly producing an increased effect [5, 6]. Furthermore, mental health problems are common in AUD patients, and nicotine may often be used as a sort of self-medication to relieve psychiatric symptoms [1]. Smoking also counters the sedative and cognitive effects of alcohol and decreases the withdrawal symptoms [3]. Smoking may also act as a gateway drug to AUD and other SUDs and be part of a drug-taking culture as alcohol increases the urge to smoke due to its disinhibiting effects [7].

The effect of smoking on AUD treatment shows that alcohol users who quit smoking in the first year after treatment were two to three times more likely to stay abstinent 9 years later [8]. Concurrent treatment for tobacco and other drugs is recommended and a meta-analysis of randomized controlled trials found that patients who were treated concurrently were 25% more likely to achieve long-term abstinence from alcohol than those not receiving a smoking intervention [9]. Smoking can trigger relapse to alcohol and similarly, relapse to smoking is more likely when alcohol use is continued [10].

As early as 1976, Green and Levy [11] stated, “It is practically impossible to cure an alcoholic (or problem drinker) so long as he continues to smoke”. Although this issue is still debatable, research has not provided an answer as to the role of smoking in the pursuit of abstinence from alcohol in patients with AUD [12]. It is important to continue research on smoking in patients with AUD and other SUDs, as smoking is becoming a more marginal phenomenon, with high use only in certain groups, e.g. persons with SUD. Many countries have now barred smoking in public places including general hospitals in an effort to curtail smoking, but addiction and mental health treatment centers seem to remain an exception to this trend [1]. Most previous studies point to the fact that smoking and alcohol or other substance addiction is a detrimental combination and that we need more research on how smoking effects the AUD/SUD treatment. The aim of this study among inpatients admitted to substance abuse treatment is therefore to estimate the prevalence of smoking and study baseline characteristics and 6 months follow-up changes in mental health and quality of life among smokers and non-smokers.

## Materials and methods

### Study design

This was an observational follow-up study using data collected at baseline both as cross-sectional data and as explanatory variables in the follow-up part of the study. The baseline data were collected within the first week of entry to the clinics, and the follow-up data at six-week and six-month follow-up. During an interview conducted by trained staff at baseline, we obtained information about mental health, substance dependence, alcohol-related variables and trauma background. Mental distress and quality of life measures were collected using self-report forms and smoking status was obtained in an interview setting at all three time points.

### Study location

Data were collected from inpatients in three different rehabilitation clinics in Eastern Norway. These clinics offer treatment for patients with various substance use problems, mainly AUD, and the length of treatment stays varies from three to 9 months.

### Study participants and inclusion criteria

Altogether 366 patients were admitted to treatment in the clinics during our inclusion period, of whom 238 (65%) were considered eligible for participation in the study. Exclusion criteria were psychosis, cognitive impairment or severe physical illness, as well as inability to speak a Nordic language. The eligible patients were provided with information about the study and 128 (54%) patients signed written informed consent.

### Ethical considerations

The study was approved by the Norwegian Regional Ethics Committee (ref. no. 2017/1314). We ensured that all methods were used in accordance with relevant guidelines and regulations.

#### Measures

##### Smoking

When we collected data about smoking, the participants were first asked: “Do you smoke cigarettes?” Those who confirmed smoking were then asked “How often do you smoke?” with the response options of ‘Daily’ or ‘Occasionally’. In the following, *smokers* refers to daily smokers, whereas *non-smokers* refers to those who do not smoke cigarettes at all or only occasionally. By comparing smoking status between time points, we counted patients who quit smoking during the baseline/six-week follow-up interval and during the six-week/six-month follow-up interval, and similarly, those who started smoking during these intervals.

##### Socio-demographic data

Information about age and sex was obtained during an interview performed at baseline, whereas educational level and employment status were reported in self-report forms collected at baseline.

##### Mental health outcome variables

The Mini International Neuropsychiatric Interview (M.I.N.I.) was used to diagnose AUD and other SUD, in addition to PTSD, current anxiety and lifetime depression [13]. Anxiety included panic disorder, agoraphobia, social phobia, and generalized anxiety disorder. The patients were asked about their history of suicide attempts. Information about traumatic experiences in childhood and adulthood was collected by means of a five-item structured self-report form. The three first questions targeted childhood trauma and covered sexual abuse, physical abuse, and other traumatic events with significant consequences, while the two last questions covered sexual and physical abuse and other traumatic events during adulthood. The response alternatives for each item were 0) ‘None’ 1) ‘Yes, once’ and 2) ‘Yes, several times’, and dichotomous variables (‘Yes’/‘No’) for childhood and adulthood trauma were constructed.

##### Mental distress

The Hopkins Symptom Checklist 10 question version (HSCL-10) is a widely used self-report tool that covers common symptoms of anxiety and depression experienced during the preceding week [14]. Each item has four ordinal responses, ranging from 1) ‘Not at all’ to 4) ‘Extremely’. The average score for each individual was calculated yielding a score between 1 and 4, where a higher score corresponded to more mental distress.

##### Quality of life

We used a five-item Quality of life (QoL-5) instrument to measure patient satisfaction with life in general [15]. This tool targets self-perceived quality of mental and physical health, and relationship to oneself and to significant others. There are five ordinal response alternatives ranging from 1) ‘Very good’ to 5) ‘Very poor’. The raw scores are transposed and inverted into a total score between 10 and 90, where a higher score indicates better life quality.

##### Treatment drop-out

Treatment drop-out was defined as discontinuation of treatment stay by leaving the clinic before the planned completion. Planned duration of treatment stay varied between patients and clinics from three to 9 months. The final follow-up of the study was at 6 months and patients still in treatment at this time point were regarded as non-drop-outs. Three patients discontinued treatment for other reasons and were not included in the analysis of the drop-out rate.

##### Alcohol-related measures

The Alcohol Use Disorders Identification test (AUDIT) was used to measure harmful drinking of alcohol. This is a 10-question instrument with responses dealing with use of alcohol during the preceding year [16]. All items have responses ranging from 0 to 4, which are added up to a total score between 0 and 40, where a higher score indicates more problematic alcohol use. In addition to the AUDIT, we asked the participants about their age at first drink and whether their parents had problems with alcohol.

##### Physical activity

Physical activity was measured with the International Physical Activity Questionnaire short version (IPAQ-S) [17, 18]. Patients reported all activities during leisure time, work, domestic activities, and transport during the preceding week. The number of days and minutes spent doing vigorous exercise, moderate exercise, walking or sitting was specifically reported in seven questionnaire items. Based on this information the patients were categorized into three levels of physical activity: low, moderate or high [19]. For the purpose of this study we dichotomized the variable into low vs. moderate/high.

##### The adult ADHD self-report questionnaire

To measure current ADHD (attention deficit hyperactivity disorder) symptoms, we used the six-item version of the Adult ADHD Self-Report Scale (ASRS), which has demonstrated good specificity and sensitivity [20]. The six items target inattentiveness and hyperactivity symptoms and there are five ordinal response alternatives ranging from 0) ‘Never’ to 4) ‘Very often’. We calculated the total score of the six items, using a cut-off point of ≥14 [21].

### Statistical analyses

Statistical analyses were performed using STATA version 15. At baseline we had data for 128 individuals for smoking, socio-demographics and most mental health variables. Thirty-one persons did not return their questionnaire (including HSCL-10, QoL, ASRS and AUDIT) and are missing in the analyses where these forms were used. The means were imputed for two persons for the HSCL-10 and one person for the AUDIT who had < 20% missing items on these measures.

We used medians and inter-quartile percentiles to describe the samples and non-parametric tests to test group differences. Unadjusted and adjusted linear regression models were used to test the associations between smoking and mental distress and between smoking and quality of life, while logistic regression was employed to test the association between smoking and treatment drop-out. The model is only adjusted for age and sex due to small sample size and low power (66 participants at 6 months).

## Results

Of the patients who declined participation, 76 were men (69%; mean age 48.7, standard deviation (SD) 11.6) and 34 were women (31%; mean age 46.3, SD 11.5). There were no significant differences between included and excluded individuals regarding sex or age.

Table 1 shows the differences in sociodemographics, mental health and alcohol- related variables at baseline between smokers and non-smokers. There were no significant differences in sex or age between the groups. Non-smokers had significantly higher educational levels in terms of both secondary education and college/university (*p* = 0.002 and *p* < 0.001, respectively).

**Table 1:** Demographics and health variables stratified by smoking habits among the patients included (*N* = 128) at baseline

		Non-smoker	Smoker	*p*
		*n* = 32	*n* = 96	
**Demographics**				
Sex (female)	n (%)	11 (34)	23 (24)	0,248^e^
Age (years)	Median (IQR^d^)	49 (37–58)	53 (44–57)	0.411^f^
13 years of education	n (%)	23 (96)	44 (63)	**0.002**^e^
16 years of education	n (%)	16 (62)	9 (13)	**< 0.001**^e^
In paid work	n (%)	6 (23)	12 (16)	0.451^e^
> low physical activity level^a^	n (%)	14 (61)	24 (36)	**0.041**^e^
**Mental health background**				
ASRS score > cutoff	n (%)	11 (44)	33 (46)	0.831^e^
Anxiety^b^	n (%)	15 (48)	64 (67)	0.068^e^
Depression, lifetime	n (%)	24 (77)	71 (74)	0.700^e^
Childhood trauma	n (%)	18 (72)	52 (72)	0.983^e^
Adulthood trauma	n (%)	17 (68)	47 (65)	0.805^e^
PTSD	n (%)	4 (13)	20 (21)	0.296^e^
Suicide attempt	n (%)	4 (13)	33 (34)	**0.018**^e^
**Alcohol-related variables**^c^				
Parent with alcohol problems	n (%)	16 (57)	45 (53)	0.699^e^
Age first drink (years)	Median (IQR^d^)	16 (15–17)	15 (13–16)	**0.028**^f^
AUDIT score	Median (IQR^d^)	28 (26–34)	30 (24–34)	0.874^f^
**Substance dependence**				
Alcohol	n (%)	28 (88)	85 (89)	0.874^e^
Other substance	n (%)	9 (28)	28 (29)	0.910^e^
Alcohol and other substance	n (%)	5 (16)	17 (18)	0.787^e^
**Treatment clinic**				
Clinic 1	n (%)	21 (66)	40 (42)	0.058
Clinic 2	n (%)	6 (19)	26 (27)	
Clinic 3	n (%)	5 (16)	30 (31)	

^a^International physical activity questionnaire short version (IPAQ-S), low vs. moderate/high level. ^b^Anxiety: panic disorder, agoraphobia, social phobia or generalized anxiety disorder. ^c^Alcohol-dependent patients only (*n* = 113). ^d^Inter-quartile percentiles (25 and 75%). ^e^Chi-square tests. ^f^Mann-Whitney U tests

Non-smokers were more physically active than smokers (*p* = 0.037). Non-smokers had had fewer suicide attempts (*p* = 0.018) than smokers, and there was a trend towards fewer patients with an anxiety diagnosis among non-smokers (Table 2). For the other mental health-related variables, there were no differences between smokers and non-smokers, including childhood and adulthood trauma and PTSD, lifetime depression and ASRS score above cut-off indicating ADHD diagnosis. There was a difference in age at first drink, where smokers had an earlier debut than non-smokers (15 versus 16 years of age, *p* = 0.028). There were no differences in AUDIT scores, whether they had a parent with problem drinking or other substance dependencies.

**Table 2:** Smoking habits at baseline, and at six weeks and six months follow-ups

		Baseline	Six weeks	Six months
		*n* = 128	*n* = 100	*n* = 66
**Non-smoker**	n (%)	32 (25)		
Continuing non-smoker	n (%)		23 (23)	18 (27)
Quit smoking	n (%)		6 (6)	6 (9)
**Smoker**	n (%)	96 (75)		
Continuing smoker	n (%)		70 (70)	41 (62)
Started smoking	n (%)		1 (1)	1 (2)

Study participants were staying at three different treatment locations. The average age ranged from 48.3 to 51.8 years in the three clinics. There were 44% females in one of the clinics, 19% in the other and the third was restricted to men only. The clinic with most women had the highest number of non-smokers (Table 1).

There were 96 (75%) daily smokers and 32 (25%) non-smokers at baseline (Table 2). One patient started smoking, and nine patients quit smoking during the study period of 6 months, six before 6 weeks and three after 6 weeks. Three of the six that quit early dropped out before 6 months so at 6 months there were still six quitters.

**Fig. 1:**
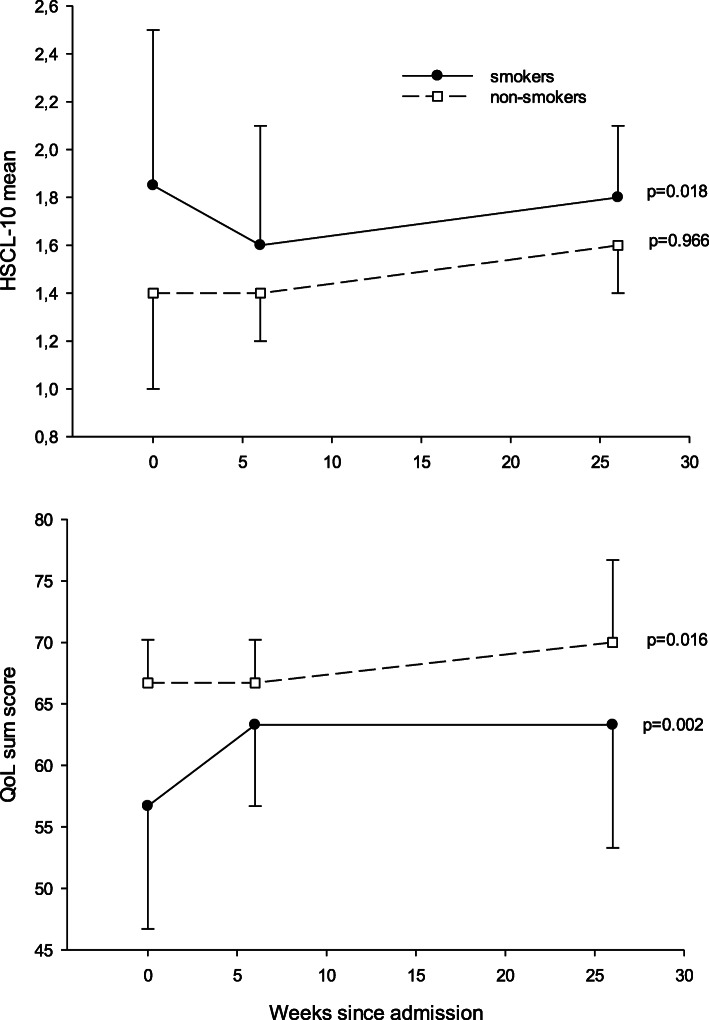
Development of mental distress and life quality during treatment period. Notes: Mental distress (HSCL-10) score (upper panel) and quality of life (QoL) score (lower panel) for smokers vs. non-smokers at baseline, 6 weeks and 6 months follow-ups. Medians and 25% or 75% percentiles are presented. Friedman tests were used to test differences across time. Complete cases are included (*n* = 45)

Table 3 shows baseline, 6 weeks and 6 months follow-up of mental distress, quality of life and drop-out from treatment. Non-smokers had fewer symptoms of mental distress (HSCL-10) at baseline than smokers (1.8 versus 2.2, *p* = 0.044). In addition, at 6 weeks and 6 months follow-ups non-smokers tended to have lower distress scores than smokers, but the differences were not statistically significant. There were similar findings for life quality with better QoL scores at baseline, though not statistically significant (*p* = 0.062), but at 6 months QoL scores in favor of non-smokers were significantly higher (70 versus 63, *p* = 0.037). The numbers of patients who dropped out of treatment after 6 months was four (13%) among the non-smokers and 35 (37%) among the smokers (*p* = 0.015), whereas at 6 weeks there was no difference between the groups. Figure 1 also shows this, but includes only those 45 participants with complete data for all three measurement points. The main difference in the figure is that smokers show a significant drop in HSCL score compared to non-smokers at 6 weeks.

**Table 3:** Levels of mental distress and life quality scores at baseline and follow-ups, and drop-out rate at 6 month follow-up

		Non-smoker	Smoker	*p*-value
**HSCL-10**				
Baseline (*n* = 97)	Median (IQR^a^)	1.8 (1.4–2.2)	2.1 (1.5–2.6)	**0.044**^b^
6 weeks (*n* = 73)	Median (IQR^a^)	1.6 (1.3–2.3)	1.9 (1.4–2.3)	0.680^b^
6 months (*n* = 45)	Median (IQR^a^)	1.6 (1.4–1.7)	1.8 (1.5–2.1)	0.253^b^
**QoL**				
Baseline (*n* = 97)	Median (IQR^a^)	63 (53–70)	53 (43–63)	0.062^b^
6 weeks (*n* = 73)	Median (IQR^a^)	63 (50–70)	63 (53–70)	0.802^b^
6 months (*n* = 45)	Median (IQR^a^)	70 (67–77)	63 (53–70)	**0.037**^b^
**Treatment drop-out**				
6 weeks (*n* = 125)	n (%)	3 (10)	10 (11)	0.934^c^
6 months (*n* = 125)	n (%)	4 (13)	35 (37)	**0.015**^c^

*HSCL-10* Hopkins Symptoms Check-list 10, *QoL* Quality of Life questionnaire

^a^Inter-quartile percentiles (25 and 75%). ^b^Mann-Whitney U tests. ^c^Chi-square test statistics

There were no significant differences in baseline characteristics between patients that quit smoking (*n* = 9) and those who continued smoking during the treatment period, either in demographics, psychiatric conditions, alcohol-related measures or mental distress and quality of life (not shown in table). Those who quit smoking reported improved quality of life during the stay, matching the levels of non-smokers at 6 weeks and 6 months follow-up (Median 57 (interquartile percentiles 48–67) at baseline, 67 (57–79) at 6 weeks and 70 (63–70) at 6 months). There was a corresponding decrease in symptoms of mental distress (1.9 (1.2–2.1) at baseline, 1.6 (1.1–2.6) at 6 weeks and 1.1 (1.0–1.9) at 6 months). Due to the small numbers involved, we were unable to make comparisons with the rest of the sample.

Table 4 shows bivariate and adjusted linear regression models of the effect of daily smoking on mental distress and quality of life at baseline and bivariate and adjusted logistic regression models of the effect of daily smoking on treatment drop-out status at 6 months follow-up. Smoking was associated with mental distress with higher baseline HSCL-10 scores for smokers after adjusting for age and sex (beta 0.48, *p* = 0.002). There was a significant adjusted association between smoking and quality of life (beta -6.91, *p* = 0.050). Smoking was associated with drop-out status at 6 months follow-up after adjustment (OR 3.76, *p* = 0.023).

**Table 4:** Bivariate and adjusted linear regression models of effect of daily smoking on HSCL-10 and QoL at baseline and bivariate and adjusted logistic regression model of effect of daily smoking on treatment drop-out during 6 months

		HSCL-10				QoL				Drop-out			
		Unadjusted		Adjusted		Unadjusted		Adjusted		Unadjusted		Adjusted	
	Reference category	β	*p*	β	*p*	β	*p*	β	*p*	OR	*p*	OR	*p*
Sex	Male	0.63	**< 0.001**	0.70	**< 0.001**	−3.34	0.350	−4.47	0.216	0.89	0.792	1.05	0.922
Age	Continous variable	−0.01	0.247	−0.01	0.202	0.08	0.587	0.09	0.531	1.01	0.414	1.01	0.480
Smoking	Non-smoker	0.34	**0.035**	0.48	**0.002**	−6.01	0.083	−6.91	0.050	3.79	**0.021**	3.76	**0.023**

*HSCL-10* Hopkins’ Symptoms Check-list 10, *QoL* Quality of Life questionnaire. Adjusted models includes all three covariates

## Discussion

The main finding from this study of SUD (mainly AUD) patients receiving inpatient treatment were that compared to non-smokers, smokers had higher drop-out rates, significantly more mental distress at baseline, and significantly lower quality of life at 6 months follow-up. The finding on drop-out rate is in line with a German study showing that smoking status at the beginning of alcohol abstinence among alcohol-dependent patients was associated with drinking outcomes where smokers had a significantly higher risk of relapse to alcohol drinking than non-smokers within the first 12 months after detoxification [12]. This finding was corroborated by Weinberger et al. [10], who found the same pattern in a national epidemiological study in the USA among readmitted patients with AUD. They showed that cigarette smoking at baseline was significantly associated with a greater likelihood of alcohol abuse and dependence at 3 years follow-up compared to non-smokers. The associations remained significant after adjusting for demographics, psychiatric disorders, other substance use disorders, AUD severity, and criteria for nicotine dependence.

Drop-out rate is a major issue in addiction therapy. In our sample, 10% dropped out of treatment before 6 weeks and 31% before 6 months. A number of studies have investigated possible factors contributing to drop-out. In a comprehensive meta-analysis, Brorson et al. found that cognitive deficits, low treatment alliance, personality disorder, and younger age were predictive of drop-out [22]. In a more recent meta-analysis, Lappan et al. found that drop-out rates also increased with a higher number of cigarettes per day at intake [23]. This is not directly comparable with our study as we have investigated the effect of abstinence from tobacco.

Another finding from this study is that there is still a very high proportion of current daily smokers among inpatients for alcohol dependence treatment compared to the rest of the population. Our finding of 75% smokers among patients in treatment for alcohol or other substance dependence is in line with a study from Australia reporting 61% smokers among AUD patients in the general population and 77% smokers among AUD patients in treatment in 2007 [3]. It also agrees with a more recent study from Germany where 75% of patients in alcohol detox treatment were current smokers at intake [12]. In an epidemiological study by Weinberger et al. comparing smoking cessation rates in the past 12 years, the authors found that the cessation rate for persons with AUD was approximately half that of persons without AUD [24].

Smokers had significantly poorer mental distress scores at baseline, but improved their scores during the six-month period, probably due to the general treatment effect. The poorer mental health status of the smokers was confirmed by their higher incidence of suicide attempts. This has been confirmed in a study by Jung et al., showing higher suicide rates among those addicted to both nicotine and alcohol compared with addiction to only one substance [25]. Both smokers and non-smokers improved their QoL scores during the treatment period, but smokers had lower QoL scores at all times and significantly lower than non-smokers at 6 months. Several studies have shown an association between lifestyle factors and life quality [26].

There are also other possible reasons why the quality of life and mental health status of smokers are lower than those of non-smokers. Persons with SUDs are more likely to suffer from financial and social hardship and with increasing taxation, the use of cigarettes may consume a substantial part of their monthly expenses, making it increasingly difficult to afford food and shelter. In addition to severe financial hardship, there is societal stigma associated with smoking, leading to challenges in integration into communities and workplaces that no longer tolerate smoking. This comes in addition to the stigma connected to mental health and addiction problems [27].

There seemed to be no difference in levels of harmful drinking among smokers and non-smokers. A kind of threshold effect could explain this lack of difference, levelling off differences at start of treatment as severity of alcohol addiction is the main criteria for admission. The difference is, however, visible in age of onset of drinking as the average age was 16 for non-smokers and 15 for smokers. Smoking is known to be a risk factor for early start and heavy drinking [28]. Another interesting difference between smokers and non-smokers was that non-smokers were more physically active at baseline. It is difficult to know whether physical activity helped participants quit smoking or whether smoking reduced physical activity.

What conclusions can we draw in terms of treatment? From our findings and the possible underlying mechanisms described above, alcohol and tobacco addiction should be treated in an integrated manner. In a recent meta-analysis, smoking cessation treatment and smoking abstinence increased the chance of abstinence from alcohol and/or illicit drugs. All studies were rated as being of strong or moderate quality by the investigators [29]. According to several alcohol treatment guidelines, including those in Norway, smoking cessation treatment should be offered to all people with SUD and the recommendations are similar to those for smokers without SUD [30]. One important message is that SUD patients are also able to quit smoking. Although the cessation rate is lower than in the general population, it is still at around 20% [31].

There are some limitations to this study. The current work is based on a mixed sample of which about nine in ten have AUD and the others have other SUDs. However, these were distributed evenly across smoking groups. In addition to the patients who completed their treatment stay before 6 months, those who were still in treatment at 6 months follow-up were regarded as non-drop-outs, raising the possibility of miscategorizing some patients. A larger sample size would have made it possible to better characterize patients who succeeded in quitting smoking and reduced the risk of false negatives.

## Conclusion

Smoking seems to be an important factor in the treatment of substance use disorders as smoking at baseline is associated with mental distress, quality of life and drop-out rate. The results indicate that smoking cessation may be recommended as an integral part of alcohol abuse treatment both before and during inpatient treatment to reduce drop-out and even improve the outcomes of mental health and quality of life.

## Data Availability

The datasets used and/or analyzed during the current study are available from the corresponding author on reasonable request.

## Abbreviations

AUD: Alcohol use disorder
SUD: Substance use disorder
QoL: Quality of life
ADHD: Attention Deficit Hyperactivity Disorder
ASRS: Adult ADHD Self-Report Questionnaire
M.I.N.I: Mini International Neuropsychiatric Interview
AUDIT: Alcohol Use Disorders Identification test
HSCL-10: Hopkins Symptom checklist-10

## References

[1] Lien L (2016). Smoking, mental health and addiction: a review of the literature. J Addicti Prev Med.

[2] Tobacco, alcohol and other drugs [https://www.ssb.no/en/helse/statistikker/royk/aar]. Accessed 5 Aug 2020.

[3] Mendelsohn CP, Wodak A (2016). Smoking cessation in people with alcohol and other drug problems. Aust Fam Physician.

[4] Kelly PJ, Baker AL, Deane FP, Kay-Lambkin FJ, Bonevski B, Tregarthen J (2012). Prevalence of smoking and other health risk factors in people attending residential substance abuse treatment. Drug Alcohol Rev.

[5] Hancock DB, Markunas CA, Bierut LJ, Johnson EO (2018). Human genetics of addiction: new insights and future directions. Curr Psychiatry Rep.

[6] Funk D, Marinelli PW, Lê AD (2006). Biological processes underlying co-use of alcohol and nicotine: neuronal mechanisms, cross-tolerance, and genetic factors. Alcohol Res Health.

[7] Biederman J, Monuteaux MC, Mick E, Wilens TE, Fontanella JA, Poetzl KM, Kirk T, Masse J, Faraone SV (2006). Is cigarette smoking a gateway to alcohol and illicit drug use disorders? A study of youths with and without attention deficit hyperactivity disorder. Biol Psychiatry.

[8] Tsoh JY, Chi FW, Mertens JR, Weisner CM (2011). Stopping smoking during first year of substance use treatment predicted 9-year alcohol and drug treatment outcomes. Drug Alcohol Depend.

[9] Prochaska JJ, Delucchi K, Hall SM (2004). A meta-analysis of smoking cessation interventions with individuals in substance abuse treatment or recovery. J Consult Clin Psychol.

[10] Weinberger AH, Platt J, Jiang B, Goodwin RD (2015). Cigarette smoking and risk of alcohol use relapse among adults in recovery from alcohol use disorders. Alcohol Clin Exp Res.

[11] Green HI, Levy MH. Drug misuse... human abuse: New York: Dekker; 1976.

[12] Hufnagel A, Frick U, Ridinger M, Wodarz N (2017). Recovery from alcohol dependence: do smoking indicators predict abstinence?. Am J Addict.

[13] Lecrubier Y, Sheehan DV, Weiller E, Amorim P, Bonora I, Harnett Sheehan K, Janavs J, Dunbar GC (1997). The MINI international neuropsychiatric interview (MINI). A short diagnostic structured interview: reliability and validity according to the CIDI. Eur Psychiatry.

[14] Derogatis LR, Lipman RS, Rickels K, Uhlenhuth EH, Covi L (1974). The Hopkins Symptom Checklist (HSCL): a self-report symptom inventory. Behav Sci.

[15] Lindholt JS, Ventegodt S, Henneberg EW (2002). Development and validation of QoL5 for clinical databases. A short, global and generic questionnaire based on an integrated theory of the quality of life. Eur J Surg.

[16] Saunders JB, Aasland OG, Babor TF, de la Fuente JR, Grant M (1993). Development of the Alcohol Use Disorders Identification Test (AUDIT): WHO Collaborative Project on Early Detection of Persons with Harmful Alcohol Consumption--II. Addiction (Abingdon, England).

[17] Craig CL, Marshall AL, Sjöström M, Bauman AE, Booth ML, Ainsworth BE, Pratt M, Ekelund U, Yngve A, Sallis JF (2003). International physical activity questionnaire: 12-country reliability and validity. Med Sci Sports Exerc.

[18] Kurtze N, Rangul V, Hustvedt BE (2008). Reliability and validity of the international physical activity questionnaire in the Nord-Trøndelag health study (HUNT) population of men. BMC Med Res Methodol.

[19] Guidelines for the data processing and analysis of the “International Physical Activity Questionnaire” [https://sites.google.com/site/theipaq/]. Accessed 5 Aug 2020.

[20] Kessler RC, Adler L, Ames M, Demler O, Faraone S, Hiripi E, Howes MJ, Jin R, Secnik K, Spencer T (2005). The World Health Organization Adult ADHD Self-Report Scale (ASRS): a short screening scale for use in the general population. Psychol Med.

[21] Kessler RC, Adler LA, Gruber MJ, Sarawate CA, Spencer T, Van Brunt DL (2007). Validity of the World Health Organization Adult ADHD Self-Report Scale (ASRS) screener in a representative sample of health plan members. Int J Methods Psychiatr Res.

[22] Brorson HH, Ajo Arnevik E, Rand-Hendriksen K, Duckert F (2013). Drop-out from addiction treatment: a systematic review of risk factors. Clin Psychol Rev.

[23] Lappan SN, Brown AW, Hendricks PS (2020). Dropout rates of in-person psychosocial substance use disorder treatments: a systematic review and meta-analysis. Addiction (Abingdon, England).

[24] Weinberger AH, Gbedemah M, Goodwin RD (2017). Cigarette smoking quit rates among adults with and without alcohol use disorders and heavy alcohol use, 2002-2015: a representative sample of the United States population. Drug Alcohol Depend.

[25] Jung M (2019). The relationship between alcohol abuse and suicide risk according to smoking status: a cross-sectional study. J Affect Disord.

[26] Al-Rubaye AKQ, Johansson K, Alrubaiy L (2020). The association of health behavioral risk factors with quality of life in northern Sweden-a cross-sectional survey. J Gen Fam Med.

[27] Schroeder SA, Morris CD (2010). Confronting a neglected epidemic: tobacco cessation for persons with mental illnesses and substance abuse problems. Annu Rev Public Health.

[28] Grucza RA, Bierut LJ (2006). Cigarette smoking and the risk for alcohol use disorders among adolescent drinkers. Alcohol Clin Exp Res.

[29] Secades-Villa R, Aonso-Diego G, García-Pérez Á, González-Roz A (2020). Effectiveness of contingency management for smoking cessation in substance users: a systematic review and meta-analysis. J Consult Clin Psychol.

[30] The Norwegian Directorate of Health (2018). Nasjonal plan for implementering av pakkeforløp for psykisk helse og rus 2018–2020.

[31] Raich A, Pinet C, Ballbè M, Mondon S, Tejedor R, Arnau A, Fernández E (2018). Multimodal treatment for smoking cessation with varenicline in alcoholic, methadone-maintained, and psychotic patients: a one-year follow-up. Tob Induc Dis.

